# Radiofrequency Ablation versus Transarterial Chemoembolization for Hepatocellular Carcinoma within Milan Criteria: Prognostic Role of Tumor Burden Score

**DOI:** 10.3390/cancers14174207

**Published:** 2022-08-30

**Authors:** Shu-Yein Ho, Po-Hong Liu, Chia-Yang Hsu, Yi-Hsiang Huang, Jia-I Liao, Chien-Wei Su, Ming-Chih Hou, Teh-Ia Huo

**Affiliations:** 1 Division of Gastroenterology and Hepatology, Min-Sheng General Hospital, Taoyuan 89502, Taiwan; 2 Department of Medical Research, Taipei Veterans General Hospital, Taipei 11217, Taiwan; 3 School of Medicine, National Yang Ming Chiao Tung University, Taipei 71150, Taiwan; 4 Department of Internal Medicine, University of Texas Southwestern Medical Center, Dallas, TX 75390, USA; 5 VA Sierra Nevada Health Care System, Reno, NV 89502, USA; 6 Division of Gastroenterology and Hepatology, Department of Medicine, Taipei Veterans General Hospital, Taipei 11217, Taiwan; 7 Institute of Clinical Medicine, National Yang Ming Chiao Tung University, Taipei 71150, Taiwan; 8 Institute of Pharmacology, National Yang Ming Chiao Tung University, Taipei 71150, Taiwan

**Keywords:** tumor burden score, radiofrequency ablation, transarterial chemoembolization, hepatocellular carcinoma, Milan criteria

## Abstract

**Simple Summary:**

Tumor burden score (TBS) has been recently introduced to assess the tumor burden in hepatocellular carcinoma (HCC), but its prognostic role in patients with early-stage HCC is unclear. We confirm that TBS is an independent prognostic predictor in HCC patients within the Milan criteria undergoing radiofrequency ablation (RFA) or transarterial chemoembolization (TACE). TACE may be an effective treatment alternative for these patients. Among patients with low TBS, RFA should be considered the priority treatment modality.

**Abstract:**

Tumor burden score (TBS), estimated by the diameter and number of tumor nodules, was recently proposed to assess the tumor burden in hepatocellular carcinoma (HCC). We aimed to evaluate the prognostic impact of TBS on HCC patients within the Milan criteria undergoing radiofrequency ablation (RFA) or transarterial chemoembolization (TACE). A total of 883 patients undergoing RFA and TACE were included. The multivariate Cox proportional hazards model was used to determine independent prognostic predictors in different patient cohorts. The TACE group had significantly higher TBS compared with the RFA group. The RFA group had better long-term survival than the TACE group in patients within the Milan criteria in univariate survival analysis. In the Cox model, serum α-fetoprotein (AFP) > 20 ng/mL, performance status 1–2, medium and high TBS, albumin–bilirubin (ALBI) grade 2 and grade 3 were independent predictors linked with mortality (all *p* < 0.001). Overall, TACE was not an independent predictor; among patients with low TBS, TACE was independently associated with decreased survival compared with RFA (*p* = 0.034). **Conclusions**: TBS is a feasible prognostic marker for HCC patients within the Milan criteria. TACE may be an effective treatment alternative for these patients. Among patients with low TBS, RFA should be considered the priority treatment modality.

## 1. Introduction

Hepatocellular carcinoma (HCC) remains one of the difficult-to-treat cancers, with approximately 906,000 new cases in 2020 globally [1]. HCC ranks fifth in incidence and the second cause of morality in males. Known risk factors for HCC include chronic infection of hepatitis B virus (HBV), hepatitis C virus (HCV), alcoholism and non-alcoholic fatty liver disease (NAFLD) [2]. According to the American Association for the Study of Liver Disease (AASLD) and European Association for the Study of Liver (EASL) management guidelines [3,4], patients with very-early- or early-stage HCC are recommended to receive liver resection, local ablation therapy or liver transplantation. Transarterial chemoembolization (TACE) and systemic treatment, mainly targeted therapy and immunotherapy, are suggested for intermediate and advanced HCC [5,6].

Radiofrequency ablation (RFA) is usually recommended for patients with small HCC within the Milan criteria. On the other hand, TACE is more suitable for multinodular nodules with adequate liver functions and performance status. Notably, for patients with early-stage HCC who are not indicated for RFA or surgical resection, TACE is the main treatment option to provide effective local tumor control [7,8,9].

Tumor burden is a crucial survival determinant in HCC. For decades, the Milan criteria (two to three nodules less than 3 cm, or single tumor ≤ 5 cm) have been the major criteria to define small HCC [10]. Traditionally, the size and number of tumor nodules are used to indicate tumor burden. However, the categorical allocation of tumor size and number may limit the statistical power in prognostic prediction. As such, several researchers suggested the paradigm shift of binary to continuous stratification to improve outcome prediction. Mazzaferro and colleagues developed the “metro-ticket” model that used continuous tumor size and number to estimate the prognosis of HCC [11]. The reason to use the metro-ticket system is that a larger tumor size and number would result in worse long-term survival (the longer the metro trip, the higher the price). Recently, Sasaki and colleagues proposed the tumor burden score (TBS) to represent the tumor size and number in cancer patients [12]. TBS is a continuous variable and has been validated in different clinical settings of HCC, demonstrating excellent prognostic estimation [13,14,15].

Patients undergoing RFA or TACE may have variable baseline characteristics, such as extent of tumor involvement, liver reserve and performance status. In addition, the comparison of long-term survival in patients with early-stage HCC undergoing TACE vs. RFA remains unclear. This study aimed to investigate the prognostic role of TBS in patients with HCC within the Milan criteria undergoing RFA or TACE.

## 2. Methods

### 2.1. Patients

Between the study period of 2002 and 2017, a total of 883 HCC patients within the Milan criteria undergoing RFA or TACE at Taipei Veterans General Hospital were prospectively enrolled and retrospectively analyzed. Their baseline characteristics, including age, sex, etiology of liver disease, performance status, tumor burden (tumor size, number, and TBS), liver functions, serum biochemistry, cancer stage and treatment, were investigated. Their survival status was inspected every 3–4 months until death or drop-out from the last follow-up. This study was approved by the institutional review board (IRB) of Taipei Veterans General Hospital (IRB protocol: 2022-01-23BC; approval date: 4 January 2022) and complies with current ethical guidelines in the Declaration of Helsinki. Waiver of patient consent was obtained and approved by the IRB due to the retrospective nature of this study.

### 2.2. Definition

HCC was diagnosed according to current clinical practice guidelines [3,16]. The performance status was defined by the Eastern Cooperative Oncology Group (ECOG) criteria [17]. Hepatitis B virus (HBV) infection was considered seropositive for hepatitis B surface antigen (HBsAg), seronegative for antibody for hepatitis C (anti-HCV), and as having no history of alcoholism. HCV-related HCC was denoted seropositive for anti-HCV, seronegative for HBsAg, and as having no history of alcoholism [18].

### 2.3. Definition of TBS

TBS was calculated as the distance from the origin of a Cartesian plane and comprised two variables: maximum tumor size (*x*-axis) and number of tumors (*y*-axis) [12,19].

TBS^2^ = (maximum tumor diameter)^2^ + (number of tumors)^2^

According to this definition, TBS was classified as three groups: low TBS (<2.56), medium TBS (2.56 to 3.94), and high TBS (>3.94).

### 2.4. Albumin-Bilirubin (ALBI) Score

The calculation of ALBI score was as follows:

ALBI score = ([log_10_ bilirubin (in µmol/L) × 0.66] + [albumin (in g/L) × −0.085])

The cut-off values of ALBI grade 1/grade 2 and ALBI grade 2/grade 3 were −2.60 and −1.39, respectively [20,21,22].

### 2.5. Treatments

Confirmed cases of HCC were discussed in the multidisciplinary cancer board for treatment recommendations. The inclusion criteria for patients with HCC are single tumor up to 5 cm or two to three nodules less than 3 cm, without vascular invasion or extra-hepatic metastasis. The contraindications of RFA are (1) tumor location (close to the pericardium, diaphragm, gallbladder, caudate lobe of liver, central bile duct and inferior vena cava), and (2) presence of large amount of ascites. The details of the RFA procedure were described previously [23]. Briefly, under local anesthesia and ultrasound guidance, RFA was performed with a 17-gauge cooled-tip electrode and the Cool-Tip radiofrequency system (Radionics, Burlington, MA, USA). Post-RFA sonography was performed to confirm that there was no immediate complication. Patients who were unsuitable for RFA or resection were suggested to receive TACE for effective tumor control. TACE was delivered according to the Seldinger procedure as described previously [24]. After RFA or TACE, serum biochemistry, AFP level, and dynamic CT scan or MRI was performed every 3 months to evaluate the treatment efficacy. Repeated RFA or TACE to eradicate viable tumors was administered if clinically indicated.

### 2.6. Statistics

Chi-squared or Fisher’s exact test was used for categorical data. The Mann–Whitney U test was used to compare continuous variables. Overall survival was assessed by the Kaplan–Meier analysis with the log-rank test. Factors that were significant in univariate survival analysis were entered into the multivariate Cox proportional hazards model to determine the independent predictors associated with survival. The IBM SPSS Statistics for Windows software, version 21.0 (IBM Corp., Armonk, NY, USA), was used for statistical analysis. A *p* value < 0.05 was considered statistically significant.

## 3. Results

### 3.1. Baseline Characteristics

**Figure 1:**
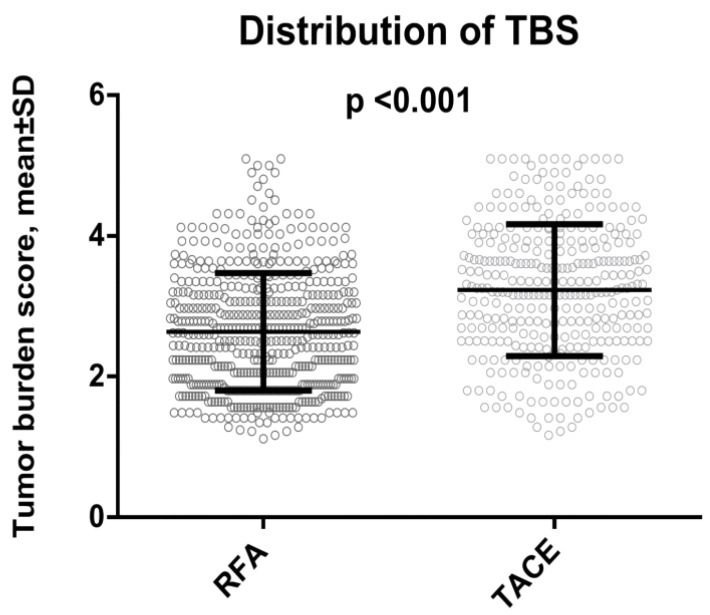
Data distribution of tumor burden score (TBS) in hepatocellular carcinoma patients within the Milan criteria undergoing radiofrequency ablation (RFA) and transarterial chemoembolization. TACE group had a significantly higher TBS compared with RFA group (*p* < 0.001).

Table 1 shows the comparison of baseline characteristics between two patient groups. The RFA group had significantly lower tumor burden (lower TBS and smaller tumors; Figure 1), better liver functional reserve, and better performance status than the TACE group (all *p* < 0.05). According to the Barcelona Clinic Liver Cancer (BCLC) stage, patients undergoing RFA more often belonged to stage 0 compared with those undergoing TACE (*p* < 0.001). No significant differences were noted in age, sex, etiology of chronic liver disease, serum α-fetoprotein (AFP), albumin, bilirubin level, and diabetes mellitus (all *p* > 0.05).

### 3.2. Kaplan–Meier Survival Analysis

The mean and median follow-up durations were 56 months and 43 months, respectively. During the follow-up, 167 (18%) patients dropped out from the study, and 627 (71%) patients died. Tumor progression and hepatic failure were the major causes of death, accounting for >95% cases.

**Figure 2:**
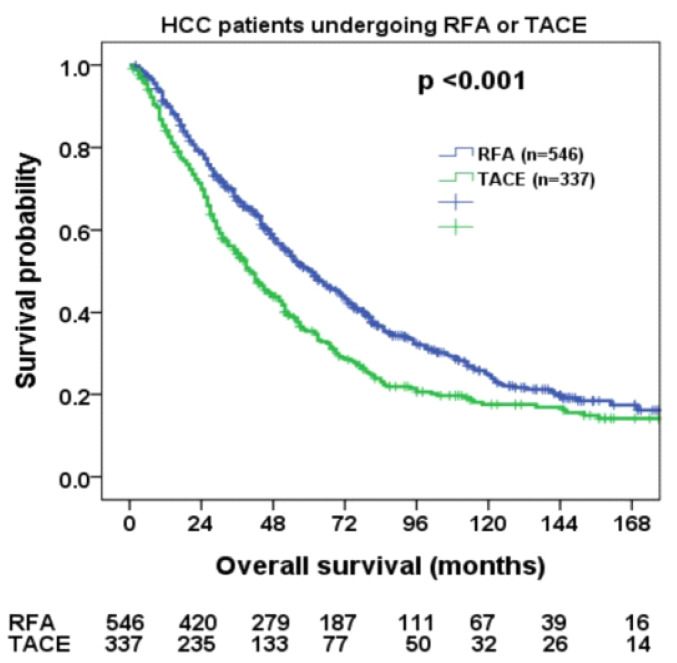
Comparison of overall survival in patients within the Milan criteria undergoing radiofrequency ablation (RFA) and transarterial chemoembolization (TACE). RFA group had a better survival compared with TACE group (*p* < 0.001).

Patients undergoing RFA had significantly longer overall survival compared with those undergoing TACE (Figure 2, *p* < 0.001); the 1-, 3-, and 5-year survival rates in the RFA and TACE groups were 79%, 59%, 17%, and 71%, 45%, 11%, respectively.

**Figure 3:**
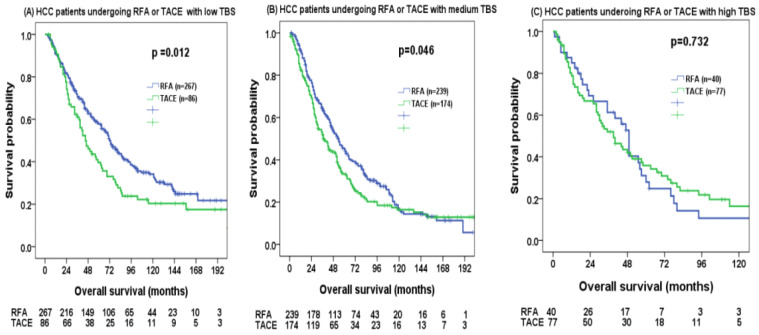
Comparison of overall survival in patients within the Milan criteria undergoing radiofrequency ablation (RFA) and transarterial chemoembolization (TACE) based on (**A**) low TBS, (**B**) medium TBS, and (**C**) high TBS. RFA group had a better survival compared with TACE group in patients with low and medium TBS (both *p* < 0.05).

We further analyzed the impact of different TBS distributions on survival. In patients with low TBS, the RFA group had a significantly better survival than the TACE group (Figure 3A, *p* = 0.012). The 1-, 3-, and 5-year survival rates for RFA and TACE groups were 82%, 64%, 22%, and 78%, 47%, 12%, respectively. In patients with medium TBS, the RFA group also tended to have a better survival than the TACE group (Figure 3B, *p* = 0.046). The 1-, 3-, and 5-year survival rates were 78%, 54%, 12%, and 70%, 44%, 10%, respectively. In patients with high TBS, there was no significant survival difference between the two groups (Figure 3C, *p* = 0.732). The 1-, 3-, and 5-year survival rates were 69%, 53%, 5%, and 67%, 44%, 12%, respectively.

### 3.3. Univariate and Multivariate Survival Analysis

For the entire cohort, age > 67 years, positive HBsAg, serum albumin < 3.5 g/dL, bilirubin > 1.1 mg/dL, ALT > 40 IU/L, platelet count < 150,000/uL, international normalized ratio (INR) > 1.0, AFP > 20 ng/mL, performance status 1–2, medium TBS, high TBS, albumin-bilirubin (ALBI) grade 2 and grade 3, and TACE were associated with decreased survival in univariate analysis (Table 2). Multivariate Cox analysis revealed that serum AFP > 20 ng/mL (hazard ratio [HR]: 1.435, 95% confidence interval [CI]: 1.221–1.687, *p* < 0.001), performance status 1–2 (HR: 1.565, 95% CI: 1.304–1.878, *p* < 0.001), medium TBS (HR: 1.372, 95% CI: 1.156–1.630, *p* < 0.001), high TBS (HR: 1.512, 95% CI: 1.181–1.937, *p* < 0.001), ALBI grade 2 (HR: 1.611, 95% CI: 1.355–1.916, *p* < 0.001), and ALBI grade 3 (HR: 2.297, 95% CI: 1.555–3.394, *p* < 0.001) were independent predictors linked with poor survival.

Patients who had low and medium TBS were subsequently analyzed to determine the role of RFA vs. TACE. In univariate analysis of the low TBS group (Table 3), age > 67 years, positive HBsAg, serum albumin < 3.5 g/dL, bilirubin > 1.1 mg/dL, ALT > 40 IU/L, platelet count < 150,000/uL, INR > 1.0, AFP > 20 ng/mL, performance status 1–2, ALBI grade 2 and grade 3, and TACE were associated with increased risk of mortality. Multivariate analysis showed that age > 67 years (HR: 1.774, 95% CI: 1.360–2.314, *p* < 0.001), platelet < 150,000/uL (HR: 1.446, 95% CI: 1.010–2.129, *p* < 0.001), ALBI grade 2 (HR: 1.738, 95% CI: 1.293–2.336, *p* < 0.001), ALBI grade 3 (HR: 2.505, 95% CI: 1.186–5.288, *p* < 0.001), and TACE (HR: 1.372, 95% CI:1.025–1.836, *p* = 0.034) were independent predictor associated with shortened survival.

In univariate analysis of the medium TBS group (Table 4), age > 67 years, serum albumin < 3.5 g/dL, bilirubin > 1.1 mg/dL, AFP > 20 ng/mL, performance status 1–2, ALBI grade 2 and grade 3, and TACE predicted a decreased survival. Multivariate Cox analysis showed that age > 67 years (HR: 1.521, 95% CI: 1.199–1.930, *p* < 0.001), serum AFP > 20 ng/mL (HR: 1.497, 95% CI: 1.189–1.885, *p* < 0.001), ALBI grade 2 (HR: 1.657, 95% CI: 1.295–2.119, *p* < 0.001), ALBI grade 3 (HR: 2.705, 95% CI: 1.602–4.570, *p* < 0.001) were independently associated with increased risk of mortality.

The analysis was further stratified according to tumor diameter. In patients with tumor size ≤ 3 cm, multivariate Cox analysis revealed that age > 67 years (HR: 1.657, 95% CI: 1.374–1.998, *p* < 0.001), positive HBsAg (HR: 1.298, 95% CI: 1.076–1.565, *p* = 0.006), serum AFP > 20 ng/mL (HR: 1.378, 95% CI: 1.144–1.661, *p* < 0.001), performance status 1–2 (HR: 1.668, 95% CI: 1.347–2.065, *p* < 0.001), medium-high TBS (HR: 1.319, 95% CI: 1.098–1.584, *p* = 0.003), ALBI grade 2 (HR: 1.738, 95% CI: 1.419–2.128, *p* < 0.001) and grade 3 (HR: 3.455, 95% CI: 2.204–5.416, *p* < 0.001) were independent predictors linked with poor survival. Among patients with tumor size > 3 cm, multivariate Cox analysis showed that serum AFP > 20 ng/mL (HR: 1.680, 95% CI: 1.233–2.290, *p* = 0.001) and ALBI grade 2–3 (HR: 1.725, 95% CI: 1.247–2.386, *p* = 0.001) were associated with decreased overall survival (Table 5).

## 4. Discussion

The Milan criteria are the major selection reference in liver transplantation for HCC. However, liver transplant in these patients is often limited by the shortage of donor organs. According to current practice guidelines, RFA is the recommended therapy for unresectable HCC within the Milan criteria. Notably, TACE is an effective treatment alternative for small HCC [25]. Very limited number of studies have specifically compared RFA vs. TACE as the primary therapeutic strategy for these patients. In this study, the long-term survival in a large patient cohort within the Milan criteria was investigated based on the distribution TBS. Our results show that although the RFA group had better long-term survival compared with the TACE group, the difference was not significant after adjustment in the multivariate Cox model, suggesting that other factors are more crucial predictors. Subgroup analysis showed that TBS is a feasible marker to discriminate long-term outcome, and we identify that TBS may provide differential impact in selecting RFA or TACE for these patients.

Tumor burden in HCC, including the diameter and number of tumor nodule as defined in the Milan criteria, is a major concern in treatment selection. Although using the categorical cut-offs is a simple and convenient way to assess disease burden, it does not appear to have clear statistical advantage, compared with continuous variables. Earlier studies proposed to use the total tumor diameter (TTD) and total tumor volume (TTV) which are also continuous scores, to assess the extent of tumor burden for HCC [26,27,28]. However, these two scores have apparent disadvantages because they require the information of size and number in all tumors during calculation. Alternatively, TBS is a more clinically feasible marker of disease burden to define the extent of tumor involvement in HCC. In our study, patients with medium and high TBS had 37% and 51% increased risk of death, respectively, compared with those of low TBS, suggesting that TBS is an important independent prognostic predictor in HCC. Consistent with previous studies [13,14,15,19,29,30], we confirm that TBS is a novel tool to discriminate survival difference patients with small HCC.

Clinically, RFA is usually recommended for patients with small HCC within the Milan criteria, and TACE is an alternative treatment option for patients unsuitable for RFA. However, the advantage of RFA over TACE in this regard is difficult to assess because the baseline characteristics are quite heterogeneous between two patient groups. In this study, we demonstrate that the survival advantage of RFA over TACE is not apparent after adjustment in the multivariate model. Notably, when the analysis was stratified according to TBS in the Cox model, we found that RFA is independently linked with increased survival compared with TACE in patients with low TBS, but not in the medium or high TBS groups. These results are consistent with previous studies [31,32], indicating that patients with low TBS are better candidates to receive RFA.

Liver functional reserve is known to play a critical role in the treatment selection for HCC. Consistently, our data indicate that patients with ALBI grade 2 and 3 had 1.6- to 2.3-fold increased risk of mortality compared with ALBI grade 1 patients [20,33,34]. Performance status is another important outcome predictor in HCC. In this study, we show that patients with poor performance status are strongly linked with decreased survival. Alternatively, the serum AFP level was reported to intimately associate with aggressive cancer behavior in HCC. Consistent with previous studies [35,36], a high AFP level is also an independent factor in predicting an unfavorable outcome.

This study has a few limitations. Firstly, in this single center study, HBV is the predominant etiology of HCC. External validation is needed from Western countries, where other etiologies are more common. Secondly, the vast majority of our patients receiving RFA or TACE did not undergo subsequent liver transplantation due to an extreme organ shortage. Therefore, our results cannot be readily interpreted in centers with a large amount of liver transplants. Lastly, a potential drawback of TBS is in its mathematical rationale that the diameter and number of tumor nodules are weighted the same in statistics.

## 5. Conclusions

In conclusion, TBS is a feasible prognostic predictor in HCC patients within the Milan criteria. TACE may be an effective treatment alternative for these patients. Among patients with low TBS, RFA should be considered the priority treatment modality. Our findings require prospective studies for validation.

## Figures and Tables

**Table 1:** Comparison of baseline demographics of patients with HCC within Milan criteria undergoing radiofrequency ablation (RFA) or transarterial chemoembolization (TACE) (n = 883).

Variables	RFA Patients (n = 546)	TACE Patients (n = 337)	*p* Value
Age (years, mean ± SD)	67 ± 11	67 ± 11	0.510
Male/Female, n (%)	351/195 (64/36)	225/112 (67/33)	0.452
Etiologies of liver disease, n (%)			0.065
HBV	234 (43)	115 (34)	
HCV	191 (35)	140 (21)	
HBV + HCV	24 (4)	19 (6)	
Others	97 (18)	63 (19)	
Performance status (0/1/2), n (%)	423/64/59 (77/12/11)	233/67/37 (69/20/11)	0.004
Diabetes mellitus, n (%)	157 (29)	99 (29)	0.843
Tumor nodules (single/multiple)	454/92 (83/17)	236/101 (70/30)	<0.001
Tumor diameter > 3 cm, n (%)	94 (17)	115 (34)	<0.001
Tumor diameter, mean ± SD	2.29 ± 0.9	2.75 ± 1.1	<0.001
Tumor burden score (TBS)			<0.001
Low	267 (49)	86 (26)	
Medium	239 (43)	174 (51)	
High	40 (8)	77 (23)	
Serum AFP (ng/mL), median (IQR)	6 (16–65)	21 (7–112)	0.142
Serum AFP ≥ 20 ng/mL, n (%)	247 (45)	171 (51)	0.112
Laboratory values, median (IQR)			
Alanine transaminase (U/L)	44 (28–74)	57 (27–74)	0.039
Albumin (g/dL)	3.8 (3.4–4.1)	3.6 (3.2–4.1)	0.744
Total bilirubin (mg/dL)	0.8 (0.5–1.2)	0.9 (0.6–1.4)	0.242
Platelets (1000/μL)	114 (86–163)	100 (71–151)	0.469
INR of prothrombin time	1.08 (1.06–1.13)	1.08 (1.01–1.16)	0.056
Creatinine (mg/dL)	0.9 (0.8–1.2)	1.0 (0.8–1.2)	0.881
CTP class (A/B)	463/83 (85/15)	265/72 (79/21)	0.019
ALBI grade (1/2/3), n (%)	242/281/23 (44/52/4)	106/217/14 (31/64/4)	0.001
BCLC stage (0/A/others), n (%)	136/290/10 (25/53/22)	32/196/109 (10/58/32)	<0.001

ALBI, albumin–bilirubin; AFP, alpha-fetoprotein; BCLC, Barcelona Clinic Liver Cancer; CTP, Child–Turcotte–Pugh; HBV, hepatitis B virus; HCV, hepatitis C virus; INR, international normalized ratio; IQR, interquartile range; SD, standard deviation.

**Table 2:** Multivariate analysis of overall survival in HCC patients within Milan criteria undergoing radiofrequency ablation or transarterial chemoembolization (n = 883).

	Univariate Analysis			Multivariate Analysis		
Overall Survival	HR	CI	*p*	HR	CI	*p*
Age (≤67/>67 years)	1.518	1.294–1.780	<0.001			
Sex (male/female)	0.936	0.794–1.103	0.432			
HBsAg (negative/positive)	1.347	1.148–1.581	<0.001			
Anti-HCV (negative/positive)	0.881	0.753–1.032	0.116			
Albumin level (≥3.5/<3.5 g/dL)	1.546	1.310–1.825	<0.001			
Bilirubin level (≤1.1/>1.1 mg/dL)	1.418	1.197–1.679	<0.001			
ALT (≤40/>40 IU/L)	1.265	1.078–1.484	0.004			
Platelet (≥150,000/<150,000/μL)	1.385	1.152–1.665	0.001			
INR of PT (≤1.0/>1.0)	1.329	1.129–1.565	<0.001			
AFP (≤20/>20 ng/mL)	1.497	1.297–1.752	<0.001	1.435	1.221–1.687	<0.001
Performance status 0/1–2	1.673	1.401–1.997	<0.001	1.565	1.304–1.878	<0.001
Tumor burden score						
Low	1			1		
Medium	1.392	1.173–1.653	<0.001	1.372	1.156–1.630	<0.001
High	1.623	1.271–2.073	<0.001	1.512	1.181–1.937	<0.001
ALBI grade						
Grade 1	1			1		
Grade 2	1.821	1.536–2.158	<0.001	1.611	1.355–1.916	<0.001
Grade 3	2.812	1.915–4.129	<0.001	2.297	1.555–3.394	<0.001
RFA vs. TACE	1.368	1.166–1.605	<0.001			

The forepart of the parentheses was defined as the reference group in the univariate and multivariate analysis. Abbreviation: AFP, α-fetoprotein; ALBI, albumin–bilirubin; ALT, alanine aminotransferase; INR of PT, international normalized ratio of prothrombin time; RFA, radiofrequency ablation; TACE, transarterial chemoembolization.

**Table 3:** Multivariate Cox analysis for RFA vs. TACE in HCC patients with low tumor burden score (n = 353).

	Univariate Analysis			Multivariate Analysis		
Overall Survival	HR	CI	*p*	HR	CI	*p*
Age (≤67/>67 years)	1.736	1.335–2.257	<0.001	1.774	1.360–2.314	<0.001
Sex (male/female)	0.952	0.730–1.243	0.719			
HBsAg (negative/positive)	1.589	1.219–2.072	0.001			
Anti-HCV (negative/positive)	0.824	0.635–1.069	0.145			
Albumin level (≥3.5/<3.5 g/dL)	1.639	1.242–2.163	<0.001			
Bilirubin level (≤1.1/>1.1 mg/dL)	1.455	1.102–1.922	0.008			
ALT (≤40/>40 IU/L)	1.457	1.111–1.911	0.006			
Platelet (≥150,000/<150,000/μL)	1.912	1.338–2.730	0.001	1.466	1.010–2.129	0.044
INR of PT (≤1.0/>1.0)	1.558	1.192–2.037	0.001			
AFP (≤20/>20 ng/mL)	1.297	1.001–1.681	0.049			
Performance status 0/1–2	1.475	1.081–2.012	0.014			
ALBI grade						
Grade 1	1			1		
Grade 2	1.983	1.496–2.628	<0.001	1.738	1.293–2.336	<0.001
Grade 3	2.329	1.122–4.835	<0.001	2.505	1.186–5.288	0.016
RFA vs. TACE	1.436	1.078–1.913	0.013	1.372	1.025–1.836	0.034

The forepart of the parentheses was indicated as the reference group in the univariate and multivariate analysis. Abbreviation: AFP, α-fetoprotein; ALBI, albumin–bilirubin; ALT, alanine aminotransferase; INR of PT, international normalized ratio of prothrombin time; RFA, radiofrequency ablation; TACE, transarterial chemoembolization.

**Table 4:** Multivariate Cox analysis for RFA vs. TACE in HCC patients with medium tumor burden score (n = 413).

	Univariate Analysis			Multivariate Analysis		
Overall Survival	HR	CI	*p*	HR	CI	*p*
Age (≤67/>67 years)	1.335	1.062–1.677	0.013	1.521	1.199–1.930	<0.001
Sex (male/female)	0.955	0.750–1.217	0.711			
HBsAg (negative/positive)	0.826	0.658–1.037	0.100			
Anti-HCV (negative/positive)	0.957	0.763–1.199	0.710			
Albumin level (≥3.5/<3.5 g/dL)	1.507	1.190–1.907	<0.001			
Bilirubin level (≤1.1/>1.1 mg/dL)	1.403	1.099–1.791	0.007			
ALT (≤40/>40 IU/L)	0.839	0.669–1.053	0.130			
Platelet (≥150,000/<150,000/μL)	0.791	0.616–1.015	0.066			
INR of PT (≤1.0/>1.0)	0.842	0.665–1.066	0.153			
AFP (≤20/>20 ng/mL)	1.526	1.218–1.913	<0.001	1.497	1.189–1.885	0.001
Performance status 0/1–2	2.024	1.573–2.605	<0.001	1.828	1.408–2.373	<0.001
ALBI grade						
Grade 1	1			1		
Grade 2	1.732	1.360–2.204	<0.001	1.657	1.295–2.119	<0.001
Grade 3	3.022	1.848–4.940	<0.001	2.705	1.602–4.570	<0.001
RFA vs. TACE	1.257	1.002–1.576	0.048			

The forepart of the parentheses was set as the reference group in the univariate and multivariate analysis. Abbreviation: AFP, α-fetoprotein; ALBI, albumin–bilirubin; ALT, alanine aminotransferase; INR of PT, international normalized ratio of prothrombin time; RFA, radiofrequency ablation; TACE, transarterial chemoembolization.

**Table 5:** Multivariate Cox analysis in HCC patients with tumor size ≤ 3 cm and >3 cm undergoing RFA or TACE.

	Univariate Analysis			Multivariate Analysis		
Overall Survival	HR	CI	*p*	HR	CI	*p*
**Tumor size ≤ 3 cm (n = 674)**						
Age (≤67/>67 years)	1.603	1.332–1.929	<0.001	1.657	1.374–1.998	<0.001
Sex (male/female)	0.998	0.826–1.208	0.987			
HBsAg(negative/positive)	1.405	1.166–1.692	<0.001	1.298	1.076–1.565	0.006
Anti-HCV (negative/positive)	0.854	0.711–1.025	0.091			
Albumin level (≥3.5/<3.5 g/dL)	1.521	1.254–1.844	<0.001			
Bilirubin level (≤1.1/>1.1 mg/dL)	1.395	1.146–1.699	0.001			
ALT (≤40/>40 IU/L)	1.353	1.119–1.635	0.002			
Platelet (≥150,000/<150,000/μL)	1.453	1.160–1.819	0.001			
INR of PT (≤1.0/>1.0)	1.324	1.097–1.599	0.004			
AFP (≤20/>20 ng/mL)	1.464	1.219–1.758	<0.001	1.378	1.144–1.661	0.001
Performance status 0/1–2	1.733	1.403–2.139	<0.001	1.668	1.347–2.065	<0.001
Tumor burden score						
Low	1					
Medium-high	1.365	1.137–1.639	0.001	1.319	1.098–1.584	0.003
ALBI grade						
Grade 1	1					
Grade 2	1.886	1.528–2.278	<0.001	1.738	1.419–2.128	<0.001
Grade 3	3.215	2.068–4.999	<0.001	3.455	2.204–5.416	<0.001
RFA vs. TACE	1.389	1.148–1.680	0.001			
**Tumor size > 3 cm (n = 209)**						
Age (≤67/>67 years)	0.861	0.627–1.182	0.335			
Sex (male/female)	1.492	1.075–2.070	0.017			
HBsAg(negative/positive)	0.894	0.652–1.225	0.485			
Anti-HCV (negative/positive)	0.910	0.663–1.248	0.557			
Albumin level (≥3.5/<3.5 g/dL)	1.650	1.190–2.287	0.003			
Bilirubin level (≤1.1/>1.1 mg/dL)	1.477	1.060–2.057	0.021			
ALT (≤40/>40 IU/L)	0.847	0.623–1.151	0.289			
Platelet (≥150,000/<150,000/μL)	1.396	1.005–1.941	0.047			
INR of PT (≤1.0/>1.0)	1.470	1.053–2.051	0.023			
AFP (≤20/>20 ng/mL)	1.717	1.261–2.339	0.001	1.680	1.233–2.290	0.001
Performance status 0/1–2	1.434	1.031–1.996	0.032			
Tumor burden score						
medium	1					
high	0.959	0.705–1.303	0.787			
ALBI grade						
Grade 1						
Grade 2–3	1.761	1.274–2.433	0.001	1.725	1.247–2.386	0.001
RFA vs. TACE	0.894	0.657–1.218	0.478			

The forepart of the parentheses was used as the reference group in the univariate and multivariate analysis. Abbreviation: AFP, α-fetoprotein; ALBI, albumin–bilirubin; ALT, alanine aminotransferase; INR of PT, international normalized ratio of prothrombin time; RFA, radiofrequency ablation; TACE, transarterial chemoembolization.

## Data Availability

The data presented in this study are available on request from the corresponding author.
